# The age-related loss of skeletal muscle mass and function: Measurement and physiology of muscle fibre atrophy and muscle fibre loss in humans

**DOI:** 10.1016/j.arr.2018.07.005

**Published:** 2018-11

**Authors:** D.J. Wilkinson, M. Piasecki, P.J. Atherton

**Affiliations:** MRC/ARUK Centre for Musculoskeletal Ageing Research and National Institute of Health Research, Biomedical Research Centre, School of Medicine, University of Nottingham, UK

**Keywords:** Muscle, Atrophy, Hypoplasia, Anabolic resistance, Denervation, Sarcopenia

## Abstract

Age-related loss of skeletal muscle mass and function, sarcopenia, is associated with physical frailty and increased risk of morbidity (chronic diseases), in addition to all-cause mortality. The loss of muscle mass occurs incipiently from middle-age (∼1%/year), and in severe instances can lead to a loss of ∼50% by the 8–9th decade of life. This review will focus on muscle deterioration with ageing and highlight the two underpinning mechanisms regulating declines in muscle mass and function: muscle fibre atrophy and muscle fibre loss (hypoplasia) – and their measurement. The mechanisms of muscle fibre atrophy in humans relate to imbalances in muscle protein synthesis (MPS) and breakdown (MPB); however, since there is limited evidence for basal alterations in muscle protein turnover, it would appear that “anabolic resistance” to fundamental environmental cues regulating diurnal muscle homeostasis (namely physical activity and nutrition), underlie age-related catabolic perturbations in muscle proteostasis. While the ‘upstream’ drivers of the desensitization of aged muscle to anabolic stimuli are poorly defined, they most likely relate to impaired efficiency of the conversion of nutritional/exercise stimuli into signalling impacting mRNA translation and proteolysis. Additionally, loss of muscle fibres has been shown in cadaveric studies using anatomical fibre counts, and from iEMG studies demonstrating motor unit loss, albeit with few molecular investigations of this in humans. We suggest that defining countermeasures against sarcopenia requires improved understandings of the co-ordinated regulation of muscle fibre atrophy and fibre loss, which are likely to be inextricably linked.

## Highlights

• Loss of muscle mass with age is due to atrophy *and* loss of individual muscle fibres.
• Anabolic resistance is fundamental in age-related fibre atrophy.
• Fibre loss is associated with denervation and remodelling of motor units.
• The plasticity of both factors should be considered in future research.

## 1 Introduction

Improvements in healthcare and nutrition have led to increased lifespan the developed world over, and thus rapid manifestation of an ageing demographic. As a consequence of the world’s ageing populace, the prevalence of chronic diseases is also on the increase e.g. since chronological age is a major pre-disposing factor to diabetes, cardiovascular/respiratory diseases, arthritic diseases and cancers (Coresh et al., 2003; Driver et al., 2008; Mitchell et al., 2012; Zierer et al., 2016). This has led to fervent efforts to develop treatments to limit the development of such chronic diseases, using pharmacological and/or lifestyle countermeasures e.g. physical activity and dietary modification. While not yet an accepted clinically diagnosable phenomena (mainly due to a lack of consensus criteria), muscle mass declines with age in a process termed sarcopenia (Mitchell et al., 2012). Longitudinal studies show that in people aged ∼75 y, muscle mass is lost at a rate of 0.64–0.7%/y in women and 0.8–0.98%/y in men (Mitchell et al., 2012). Muscle function (e.g. using strength-related performance as a proxy) is lost more rapidly, with longitudinal studies showing that at aged ∼75 y, strength is lost at a rate of 3–4% per year in men and 2.5–3% per year in women (Mitchell et al., 2012). It is beyond the scope of this review to outline the ever evolving recommendations for diagnostic criteria and prevalence of sarcopenia; instead readers are directed to detailed reviews from the European Working Group on Sarcopenia in Older People (EWGSOP) (Cruz-Jentoft et al., 2010). Nonetheless, it is clear that the major risk factors for sarcopenia are chronological age (obviously) and long-term care settings (Cruz-Jentoft et al., 2014), with the prevalence in community settings being considerably lower. Irrespective, the consequences of muscle wasting and weakness engender numerous physiological and psycho-social impacts i.e. i) inability to independently perform tasks of daily living, ii) frailty and increased risks of falls, iii) loss of independent living and related depression/social isolation, iv) physical inactivity (sedentarism), v) increased risk of chronic diseases, vi) increased risk of all-cause mortality; (Arango-Lopera et al., 2013).

Thus, arises the question: why does sarcopenia have such devastating global health effects? Clearly, frailty arises from impairments of skeletal muscles’ function to generate voluntary movement; simply put, loss of mass and function limits muscles’ fundamental capacity to generate force. However, skeletal muscle is also important in regulating whole-body metabolic health. For instance, muscle is responsible for the majority of post-prandial glucose disposal (DeFronzo et al., 1985; Shulman et al., 1990) and in supplying substrates for other tissues energy needs (e.g. glucogenic amino acids for hepatic gluconeogenesis) during fasted periods, with muscle protein stores being replenished upon intake of dietary protein (Brook et al., 2016a, 2016b). A failure in these processes can lead to perturbations in homeostasis e.g. hyperglycaemia or muscle catabolism. Moreover, physical activity, the levels of which decline with age (McPhee et al., 2016), can also protect against muscle atrophy through promotion of muscle hypertrophy/strength/fatigue resistance. On this basis, it has been suggested that maintaining physical activity with ageing is important since: i) exercise can positively influence muscle mass/function and metabolic health (notably, interventions do not always affect the trajectory of chronic diseases (Uusitupa et al., 2009)), and ii) physical activity stimulates secretion of so-called “myokines” purported to underlie many of the trans-organ health benefits of exercise (Pedersen and Febbraio, 2008) (although with little-to-no evidence of how ageing affects these functions). It is not a new observation that muscles secrete factors that act in an auto/para/endocrine manner with humorally acting “protein” factors having been suggested to influence muscle glucose uptake nearly a quarter of a century ago (Gao et al., 1994). Moreover, it is logical that cross-talk between muscle and adipose/hepatic tissues exists to regulate the established liberation of energy substrates during exercise; this is no different from the adrenal glands releasing catecholamines to promote lipolysis. Indeed, while there is much excitement in relation to myokines and how they may impact sarcopenia (and chronic diseases) - especially those targeting myostatin – results to date in relation to neuromuscular outcomes have been largely disappointing. This is perhaps unsurprising as upregulation of myostatin is likely not a feature of ageing (Ratkevicius et al., 2011; Ryan et al., 2017), and anti-myostatin therapies fail in animal models of neuromuscular decline because the myostatin pathway has already been adaptively shut-down (Saitoh et al., 2017). The remainder of this review will maintain a focus on the two key areas of age-related muscle atrophy and dysfunction and their analytical quantification in humans; namely: 1) whole-muscle and fibre atrophy and mechanisms, and 2) neuromuscular degeneration, fibre hypoplasia and their mechanisms.

## 2 Muscle fibre atrophy in humans: quantification, evidence and mechanisms

### 2.1 Quantification of MPS and MPB in humans

In order to quantify muscle protein turnover in humans to delineate the effect of ageing upon skeletal muscle homeostasis, sensitive analytical methods are required to measure MPS and MPB. This has been achieved through the application of stable isotopically labelled amino acids (AA; using 2H, 13C, 15 N, and 18O) to “trace” the movement of label from the blood and into and out of tissues and proteins. These heavy isotopes are typically distinguished from their more abundant lighter isotope by mass spectrometric techniques (effectively expensive weighing instruments). A simplified overview for those unfamiliar with these approaches follows. Traditionally, MPS is determined from muscle sampling (biopsy), extracting the tracer bound protein (the product) and measuring the amount of ‘label’ (e.g. the heavier isotope) incorporated over time in relation to the precursor, typically the enrichment of the tracer in the bloodstream/muscle over time (e.g. for ^13^C phenylalanine), or enrichment of the deamination product in the case of AA such as leucine (KIC) that are deaminated in muscle. This is the so-called “precursor-product” relationship, where the precursor labelling relates to the product labelling. In contrast, MPB is typically quantified via dilution of a tracer across a tissue, organ, or limb. This is conducted by tracking the enrichment and concentration of the tracer (usually in a steady state), ideally in the artery supplying the tissue, and measurement of its dilution in the vein draining the organ or limb. For instance, an increased venous dilution of the tracer (through AA being released from the muscle) would indicate elevated MPB (Ra- rate of appearance). It is also possible to determine net protein balance (NB) by comparing identically timed AA concentrations in the main artery/vein feeding/draining the limb/organ (as a product of arterial blood flow), which provides the “net” anabolic or catabolic state of a given limb muscle mass. NB in a steady-state is a result of the balance between the rates of MPB and MPS, therefore with measures of Ra and NB, rates of MPS (Rd- rate of disappearance) can be determined. These techniques have, and continue to, generate the majority of insight into the impact of age on human muscle proteostasis. We further refer readers to (Biolo et al., 1995b; Wilkinson, 2016; Wilkinson et al., 2017a; Wolfe and Chinkes, 2005; Zhang et al., 2002) for more in depth technical insight.

### 2.2 MPS and MPB in human ageing

**Fig. 1:**
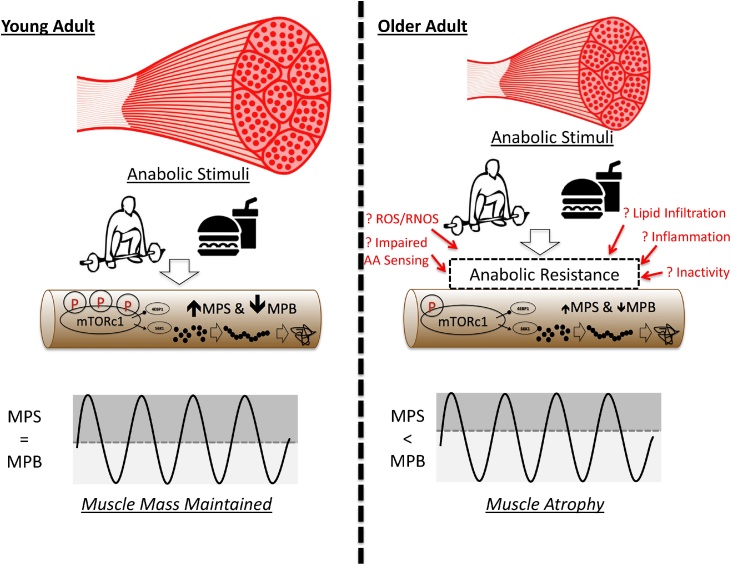
Summary of the purported mechanisms driving anabolic resistance and muscle atrophy in older age. In young adult muscle the response to anabolic stimuli such as mechanical sensing and feeding, provides stimulation of MPS and inhibition of MPB regulated primarily via control through mTORc1 signalling helping to maintain muscle mass. In older age, muscle becomes resistance to these anabolic stimuli, leading to impaired MPS and suppressed inhibition of MPB, consequently leading to the onset of atrophy. The factors driving this anabolic resistance and atrophy are not well described, however a number of theories have been proposed as highlighted in the figure and discussed within this review.

Muscle mass is regulated by the dynamic balance between MPS and MPB, with the major two environmental influences on these processes being food intake and physical activity. The intake of dietary protein influences MPS by driving the stimulation of MPS (Atherton et al., 2010a), while insulin suppresses MPB (mediated by insulinogenic AA and/or carbohydrates: (Greenhaff et al., 2008)). This acts to replenish muscle protein lost during catabolism in the fasted state (Atherton et al., 2016c) e.g. due to efflux of muscle AA to support hepatic gluconeogenesis. It has been known for over 30 years that AA represent the primary nutrient driver behind feeding induced increases in MPS (Bennet et al., 1989), with this stimulation almost exclusively driven by the essential AA’s (EAA), in particular leucine (Smith et al., 1998, 1992, Wilkinson et al., 2017b, 2013). These anabolic responses to dietary protein are both dose dependent and transient in nature, where maximal MPS responses are achieved with ≥10 g of EAA in younger individuals (Atherton et al., 2010a; Cuthbertson et al., 2005), and with exercise being able to extend the duration of this anabolic response (beyond 2–3 h) when performed alongside intake of dietary protein of free EAA mixtures (Cuthbertson et al., 2006; Miller et al., 2005; Phillips et al., 1997). This creates a situation where, across a diurnal cycle, MPS = MPB and muscle mass remains constant (Fig. 1). The importance of physical activity/movement in regulating muscle mass homeostasis is best exhibited in relation to the impact of complete immobilization (e.g. casting, bed rest) or what we would term “partial immobilization” (e.g. reduced movement due to sedentary behaviour and limited recreational activity (Atherton et al., 2016a; Breen et al., 2013a; de Boer et al., 2007a, 2007b)), which induce rapid muscle atrophy. As such, for muscle atrophy to occur, MPB must exceed MPS via a decrease in MPS and/or increase in MPB and this must to some extent be dysregulated in ageing. The search for the environmental drivers of dysregulated muscle proteostasis regulating age-related muscle loss remains hotly researched (Atherton et al., 2016b).

It follows that the burning question remains; what happens to these tightly regulated homeostatic processes in relation to age-related skeletal muscle atrophy? Ground breaking work more than 10 years ago helped to develop the idea of “anabolic resistance” to explain the phenomenon of age related muscle loss. The premise being that increases in MPS and suppressions in MPB in response to the key environmental factors regulating muscle maintenance i.e. food intake (Cuthbertson et al., 2005) and exercise (Kumar et al., 2009) are blunted compared to younger people. While this remains a contentious subject with mixed results from studies comparing young and old (Paddon-Jones et al., 2004; Symons et al., 2009, 2007), recent meta-analyses supports the existence of anabolic resistance with age (Moore et al., 2015; Wall et al., 2015). Indeed, an early study identified no age-related differences in basal/postabsorptive protein turnover (Volpi et al., 2001); but has failed to be repeated. Therefore, the potential scenarios are: i) anabolic resistance is the major driver of age-related muscle loss, ii) MPS and MPB are not key factors – which seems highly unlikely, or iii) since sarcopenia is a slow and incipient process, the aetiology cannot be captured by short-term metabolic studies over a few hours.

In relation to this latter point, a criticism of short-term tracer studies has been the ability to extrapolate the findings of acute tracer studies performed over several hours in a controlled environment to that of free living real life situations. For example, acute MPS responses to resistance exercise training (RET) do not correlate with the end-point it aims to relate to – changes in mass with chronic RET (Mitchell et al., 2015c). However recent technical developments in terms of stable isotope tracer techniques have allowed for the chronic measurement of protein turnover over days, weeks and months through the use of the stable isotope tracer deuterium oxide (D_2_O) or “heavy water”. Administered orally, the deuterium from D_2_O is incorporated onto different substrates, such as AA, at stable C–H positions through biological reduction during *de novo* synthesis, allowing rates of skeletal muscle protein turnover to be measured. Furthermore, the rapid equilibrium of D_2_O across tissue pools, combined with slow turnover of both the body water pool and skeletal muscle proteins, this technique is perfectly suited for the measurement of protein turnover over periods of days-weeks and months, overcoming the limitation of more traditional tracer techniques (Brook et al., 2015, 2016a, 2016b; Wilkinson et al., 2015, 2014). Using these techniques, the presence of anabolic resistance to the trophic effects of exercise in ageing has been confirmed, with significantly reduced rates of cumulative MPS in response to 6-weeks of unilateral RET compared to young, which accompanied blunted mass gains (Brook et al., 2016a, 2016b). Nonetheless, it remains a leap of faith to suggest that anabolic resistance to RET is responsible for age-related muscle loss. That being said, we speculate that by extension, even habitual movement, which normally acts to help maintain muscle mass (even in youth), becomes a less effective cue for muscle maintenance in older people.

In terms of the contribution played by MPB to age-related muscle loss, this has been more difficult to ascertain due to myriad technical challenges associated with measuring MPB. While techniques such as fractional break down rate (FBR) (Zhang et al., 2002) and A–V balance (Biolo et al., 1995a) can provide estimates of MPB, they rely on numerous assumptions. For instance, with A–V balance, there are assumptions of tissue specificity (i.e. that the sampled vein is only draining muscle tissues) while these methods also rely on other variable physiological measures (blood flow), that are technically challenging. Hence there is a lack of studies available on MPB and ageing. That being said, it has been observed that in older adults there is blunted inhibition of MPB in response to increases in plasma insulin equivalent to that of post-prandial levels (Wilkes et al., 2009) – i.e. an “*insulin resistance of protein metabolism*”. Therefore, it is assumed that the combination of suppressed inhibition of MPB by insulin combined with blunting of MPS (in response to dietary protein and movement), exacerbates anabolic resistance in older adults, and presumably muscle atrophy.

Another physiological driver of age-related muscle atrophy is thought to be physical inactivity, since older age is associated with moving less and abstention from recreational sporting activities (McPhee et al., 2016). Indeed, it is already well-established that either complete or partial immobilization causes muscle atrophy, irrespective of age. For example, unilateral limb immobilization leads to rapid muscle atrophy even in young healthy adults, with ∼5% reduction in muscle CSA after only 14 days (de Boer et al., 2007b) with recent evidence suggesting that much of this loss occurs within the first few days (Wall et al., 2014). This loss of mass is accompanied by anabolic resistance; vis-à-vis, decreases in both the rate of post-absorptive MPS (de Boer et al., 2007a) and a blunted response to nutrition (Glover et al., 2008). Even more strikingly, merely reducing daily activity (step count) induces anabolic resistance (Breen et al., 2013b) and causes muscle atrophy. Since older people tend to move less, we would suggest that this, coupled to age-related anabolic resistance, are key drivers of sarcopenia. Nonetheless, whether older individuals are more ‘susceptible’ to immobilization related muscle loss, remains controversial. Indeed, while one early study showed older adults losing muscle at twice the rate of younger people (Kortebein et al., 2007), others have shown lower levels of muscle mass loss in older vs. younger individuals over the same period of immobilization (∼3.5% vs ∼1.5% reduction in CSA in young and old respectively; (Dirks et al., 2014; Wall et al., 2014). This controversy aside, older adults do seem to have a reduced capacity to recover muscle loss completely, despite supervised rehabilitation (Suetta, 2017; Suetta et al., 2013). Age-related muscle atrophy, therefore, appears to be caused by a combination of behavioural and physiological interacting factors, from resistance to anabolic stimuli, leading to suppressed MPS and inhibited suppression of MPB, to inactivity and immobilization, and perhaps with an exacerbation of atrophic responses and a failure to fully recover.

### 2.3 The molecular regulation of muscle protein turnover and atrophy in human ageing

In the case of the molecular regulation of dysregulated MPS and MPB with ageing, a number of studies have aimed to delineate the sites of molecular dysregulation: i) in response to nutrition, ii) in response to exercise, and iii) in relation to the upstream drivers of these processes. In relation to the regulation of muscle nutrient sensing and signalling (and potential dysregulation in ageing) by nutrients, EAA, and in particular leucine (Atherton et al., 2010b; Wilkinson et al., 2013), while also being a substrate for MPS (i.e. a proteinogenic AA) are also a signalling molecule (Bonfils et al., 2012; Han et al., 2012; Moro et al., 2016). For example, leucyl tRNA synthetase (enzyme that attaches leucine to its cognate tRNA) binds to GTPases, known mediators of mTORc1, activating mTOR signalling (Bonfils et al., 2012; Han et al., 2012). Due to this need for both substrate and sensing, it has been proposed that reductions in AA delivery to the muscle may impair this intracellular signalling and hence MPS (Moro et al., 2016). Initially it was proposed that impaired dietary absorption, through increased splanchnic AA extraction in older adults, might contribute to reduced AA delivery to muscle (Boirie et al., 1997; Moreau et al., 2013). However, hyperaminoacideamia following large doses of protein or AA is actually higher than, and more prolonged, in older adults (Koopman et al., 2009; Mitchell et al., 2015a, 2015b), suggesting that impaired digestion/absorption of AA is not a limiting factor in MPS. Since ageing leads to reductions in limb blood flow (Skilton et al., 2005) with associated blunting of post-prandial micro and macro vascular blood flow to muscle (Mitchell et al., 2013), altered delivery of AA could still impact muscle anabolism. In support of this, work from one group showed muscle anabolism could be impaired by reducing microvascular blood flow (MVF; using NOS inhibitor L-NMMA; (Timmerman et al., 2010a)), and augmented by increasing MVF (using the NO donor SNP; (Timmerman et al., 2010b). However, more recent work has shown that enhancement of microvascular responses to feeding in older men using exercise (Phillips et al., 2015), cocoa flavanols (Phillips et al., 2016) and the NOS precursor arginine (Mitchell et al., 2017), did not improve muscle anabolism, suggesting AA delivery is unlikely a factor in age induced atrophy. This is in line with work showing that the post-prandial intracellular concentrations of AA actually tend to be higher in older individuals than young (Paddon-Jones et al., 2004), presumably due to compromised clearance by MPS. Therefore, impaired intracellular induction or propagation of mTORC1 signalling is the most likely candidate, in line with early findings of impaired mTORC1 substrate phosphorylation in older vs. younger muscle (Cuthbertson et al., 2005; Guillet et al., 2004). In contrast, nothing is known of how “*insulin resistance of protein metabolism*” is regulated; we postulate that impaired age-related insulin resistance is associated with impaired cross-talk to anti-catabolic pathways. This notion could be viewed as akin to the dysregulation of signalling between the insulin receptor and GLUT4 translocation (and thereby glucose uptake).

In terms of sensing movement/exercise, it is becoming increasingly evident that intrinsic mechano-sensitive signalling pathways act to increase mTORC1 activity post exercise (Hornberger et al., 2006; O’Neil et al., 2009). However it has been shown that p70S6K signalling is blunted in response to acute exercise in older age (Fry et al., 2011; Kumar et al., 2009), as well as being impaired temporally in response to 6-weeks RET (Brook et al., 2016a, 2016b) thus reducing translational efficiency (meaning the activity of pathways involved in co-ordinating the rate of mRNA translation). Interestingly, in the same study aspects of translational capacity; RNA content and ribosomal biogenesis, were also investigated, with blunted expression of rDNA transcription factors cMyc and TIF1a, alongside blunted increases in the indices of translational capacity (RNA:DNA and RNA:Protein ratios) being observed in the older adults in response to RET (Brook et al., 2016a, 2016b). This suggests that impaired ribosomal biogenesis and capacity for MPS in older adults may also be a key factor underlying anabolic resistance. It is not only RNA which may be impaired in the skeletal muscle of older adults. It has been posited that skeletal muscle satellite cells (SC), which provide an essential role for the regeneration and repair of muscle fibres through the provision of additional myonuclei to the mature post-mitotic muscle cells (Lepper et al., 2011), may also play an important role in muscle growth (in response to RET) and muscle mass maintenance (Snijders and Parise, 2017). With evidence of a decline in skeletal muscle SC content with age (Verdijk et al., 2014), it may be that this decline in concert with a decline in SC function with age could be a factor in the inability to maintain muscle mass (Snijders and Parise, 2017). However based on the current models used (pre-clinical models associated with genetic/irradiated ablation of SC; (McCarthy et al., 2011; Rosenblatt et al., 1994)) to assess the role of SC in skeletal muscle growth and ageing, and the continued conflicting findings arising from these experiments (Blaauw and Reggiani, 2014), it remains unclear as to the importance of SCs in the maintenance of muscle in older age and in relation to muscle hypertrophy with RET. However, with new, novel stable isotope tracer techniques available for directly measuring both RNA and DNA synthesis in muscle (Brook et al., 2017), the overall influence of ribosomal biogenesis and satellite cells in relation to anabolic resistance can also begin to be determined.

Sarcopenia is not only associated with a loss of mass, but also a loss of muscle quality, associated with an increase in extra and intra-myocellular lipid deposition (Delmonico et al., 2009). This increase in intracellular muscle lipids has been linked to the development of insulin resistance, and in turn, could impact muscle protein turnover. In relation to this, it has been shown i) that lipid induced insulin resistance in younger adults leads to a reduction in MPS in response to AA, with suppression of mTORc1 signalling (Stephens et al., 2015), and ii) that obese adults exhibit anabolic resistance in MPS to nutritional cues (Murton et al., 2015). Not only can lipid infiltration lead to insulin resistance that could relate to dysregulated proteostasis in ageing, but it has also been linked to the development of inflammation (Kalinkovich and Livshits, 2017; Rivas et al., 2016). A number of studies have identified an association between inflammatory markers and loss of muscle mass (Cesari et al., 2005; Visser et al., 2002), e.g. with a negative correlation between the inflammatory marker CRP and muscle mass being observed in older women (Wåhlin-Larsson et al., 2017). Whilst the mechanisms are unclear, studies in rats have shown that the reduction of low grade inflammation can restore post-prandial muscle anabolism (Rieu et al., 2009). Recent work has purported that CRP itself may act as a catabolic regulator in muscle by inhibiting mTORC1 via depression of upstream signalling through AKT/PI3K, and/or an increase in intracellular energy stress via upregulation of AMPK which can directly inhibit mTORc1 (Wåhlin-Larsson et al., 2017). In sum, while the upstream regulators of anabolic resistance are ill defined, dysregulated lipid handling is a candidate.

Finally, the production of reactive oxygen and nitrogen species (RONS) and oxidative damage has long been thought of as a potential mechanism of age-related muscle atrophy through the radical theory of ageing, whereby RONS damage proteins, lipids and DNA leading to dysfunction of the tissues (Harman, 1956). Moreover, an essential role has been proposed for ROS in regulating the IGF-AKT-mTOR signalling pathway (Nacarelli et al., 2015), which in turn can directly impact control of muscle protein turnover. Although much of this work has been performed in genetic pre-clinical models (e.g. knocking out genes involved in scavenging free radicals), a recent human study looking at levels of protein carbonylation - a marker of oxidative damage - in muscle showed that levels of protein carbonyls increased with age, but no difference was observed between those designated as sarcopenic and non-sarcopenic (Beltran Valls et al., 2015). Therefore the contribution of RONS to age related muscle decline remains debatable, while the use of antioxidant therapies has proved largely unsuccessful (Deane et al., 2017), and with antioxidants potentially having adverse effects on muscle (Gomez-Cabrera et al., 2008). As such, while their role is difficult to define in humans, there is little evidence that redox imbalances are key drivers of age-related muscle atrophy, nor in response human disuse atrophy (Glover et al., 2010).

In sum, whilst there are many other aspects of physiology which have been proposed to contribute to skeletal muscle atrophy with age, such as declines in mitochondrial content and function with age (Johnson et al., 2013; Rooyackers et al., 1996) and disturbances to the hormonal mileiu (Basualto-Alarcon et al., 2014), there is always going to be research striving to find this “magic bullet” compound or intervention to combat age-related skeletal muscle wasting. Therefore, more research is needed to determine the location and mechanisms of the road-blocks between the key environmental cues for muscle maintenance (i.e. movement and food) and the regulation of muscle homeostasis. Nonetheless, there is clear evidence of metabolic inflexibility in protein metabolism in older age; Fig. 1 represents a summary of the potential major drivers of age-related muscle atrophy in humans, as discussed above.

## 3 Muscle fibre loss in humans: quantification, evidence and mechanisms

### 3.1 Quantification of muscle fibre number (hypoplasia) in older humans

Beyond muscle atrophy, the second, and likely inter-connected mechanism of whole muscle atrophy is that of muscle fibre loss (hypoplasia). The gold-standard measurement is direct anatomical estimates obtained from cadaveric studies, although for obvious reasons these studies are rare. Among the first human studies of this nature was from Lexell et al (1983) who compared data from 12 cross sections of autopsied vastus lateralis (VL) of ∼30 and ∼72 year old men (Lexell et al., 1983). The mean total muscle size of the VL was 18% smaller in the old. The difference in total muscle size was purported to be accounted for by a marked reduction in the *number* of myofibres in the older muscle (478,000 vs. 364,000). The same group later expanded this evidence with a further 43 full cross sections of VL, from men aged 15–83 years, and noted a reduction in total muscle size from 20 to 80 years of 40%, largely associated with a 39% reduction in the *number* of fibres across the same age range (Lexell et al., 1988). It is worth noting that muscle fibre loss did not account for the entirety of total muscle loss, as smaller (i.e. atrophic) fibres were also observed in older muscles, in addition to the fact that there was a ∼20% greater amount of non-contractile material in the old muscle, which would artificially inflate total muscle CSA.

The nature of such detailed anatomical counts and estimates explains why they are so rare; however further studies have made estimates by dividing mean fibre CSA into total muscle CSA. In a 12-year longitudinal study of nine men, Frontera et al (2000) reported an average 14.7% decrease in quadriceps CSA (from 65 to 77 years) with no decrease in individual fibre CSA, suggesting fibre loss was responsible for the total whole muscle atrophy (Frontera et al., 2000). In relation to muscle loss and force producing capacity, Jubrias et al (1997) reported a 21% decline in total muscle size between 65–80 years, with a 39% decrease in force, equating to a 21% decline in specific force (i.e. force normalized to muscle size) (Jubrias et al., 1997). Thus, the age-related decreases in force producing capacity cannot be explained entirely by a decrease in muscle size - likely suggesting deleterious neuromuscular remodelling. Indeed, although alterations in older muscle fibres independent of size will reduce their force generating capacity (Ochala et al., 2007), denervated fibres are present in older human muscle (Spendiff et al., 2016), which contribute to total muscle size but their lacking of innervation would mean a failure to contribute to force generating capacity.

More recently, in a study estimating VL fibre number from biopsy and total muscle CSA, data from 31 young (∼22 y) and 40 old (∼72 y) men and women estimated the age-related difference in total muscle size was due, in almost equal amounts, to fibre atrophy and a reduction in the number of fibres in the old (McPhee et al., 2018). However this evidence is not equivocal, as the same methods showed the difference in fibre number in bicep brachii (BB) between young (21 ± 2 years) and old (82 ± 2 years) to be minimal (253,000 vs 234,000) (Klein et al., 2003). This discrepancy may be explained by the differential response to ageing observed in different muscles (Pannérec et al., 2016; Piasecki et al., 2018b), and the minimal age-related loss of CSA in BB (Janssen et al., 2000). Moreover, Van Loon and colleagues showed that up to 100% of age-related whole muscle atrophy in the VL could be explained by fibre atrophy without the need for fibre loss – based on MRI of thigh muscles and determining fibre area of related muscle biopsies (Nilwik et al., 2013), although older fibre CSA reported here were around 25% larger than previously reported areas (Gouzi et al., 2013). As such, it remains controversial as to the contributions of atrophy and fibre loss in whole muscle atrophy. We suggest it is highly likely taking all together that both are key factors at play in sarcopenia, and that existing data are compromised by both methodological and physiological differences.

Further indirect evidence of age-related fibre loss comes from the maximal compound muscle action potentials (CMAP) of young and old muscle, whereby motor neurons serving the muscle of interest are electrically stimulated in order to elicit a maximal contraction. The electrical activity of this contraction can be measured via electromyography (EMG) to provide an estimate of the amount of contractile material contained within the recording limits of the EMG electrode. Although not without its limitations (Piasecki et al., 2018a), this method consistently shows older muscle has a smaller CMAP than young in a range of muscles, including tibialis anterior (TA) (McNeil et al., 2005; Piasecki et al., 2016a), soleus (Dalton et al., 2008), BB (Power et al., 2012), and VL (Piasecki et al., 2016c), with a further study showing no age-related difference in TA (Hourigan et al., 2015). The assumption here is that the relatively small volume of muscle recorded from is constant, then the older muscle contains a reduced amount of contractile material, probably as a result of *fewer* and *smaller* muscle fibres, combined with an increased number of denervated fibres (see below).

Clearly, differences in experimental design, technical challenges of methodologies, variation between individual muscles, and environmental influences between subjects are all considerations leading to the apparent discrepancies between studies. Nonetheless, once again, it would generally appear that both muscle fibre atrophy and the loss of fibres are highly likely to be factors in age-related atrophy of whole muscles.

### 3.2 Quantification of motor unit number in older humans

**Fig. 2:**
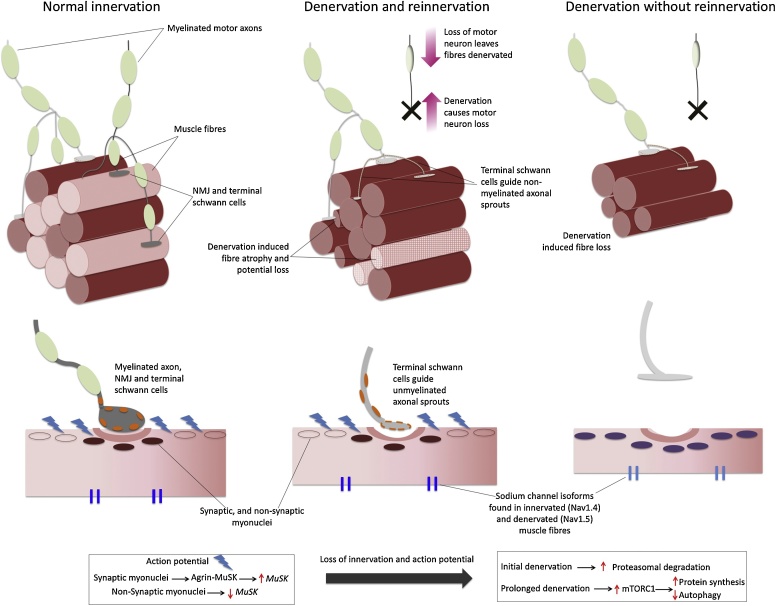
Summary of denervation induced muscle fibre hypoplasia Top: With normal innervation, myelinated axons communicate with the muscle fibre at the NMJ. Each neuron and all muscle fibres connected to it via the NMJ are part of the same MU. Denervated fibres may be reinnervated by schwann cell guided axonal sprouting, or they may atrophy and eventually be lost. Bottom: With normal innervation, electrical activity from action potentials suppress NMJ maintenance genes in non-synaptic nuclei, with expression maintained in synaptic nuclei via agrin-MuSK signalling. Immediately post denervation proteasomal degradation is increased, then decreased with prolonged denervation combined with an increase in protein synthesis.

Loss of muscle fibres is associated with the age-related loss of motor units (MU) (Faulkner et al., 2007; Hepple and Rice, 2016; Piasecki et al., 2016b). Described as the last functional unit of the motor system, the human MU comprises a cell body in the ventral horn of the spinal cord, the alpha motor neuron and all of the muscle fibres it innervates. Again, post mortem anatomical studies have provided a wealth of information on the effects of age; with there being a progressive decrease in the number of cell bodies in spinal cord sections aged over 60 years, and those aged over 75 years having 30% fewer serving the lower limbs than young (Kawamura et al., 1977; Mittal and Logmani, 1987; Tomlinson and Irving, 1977). Human *in vivo* studies utilising EMG techniques have also shown an age-related decline in MU number, in small (Galea, 1996), and larger muscles (McNeil et al., 2005; Hourigan et al., 2015; Piasecki et al., 2016a, Piasecki et al., 2016c. The loss of a MU will leave a muscle fibre denervated and more susceptible to atrophy and eventually loss. However, many fibres will be re-innervated by a nearby surviving axon. These axonal sprouts originate from non-myelinated areas of the axon and can ‘rescue’ a denervated fibre in an attempt to preserve muscle mass, termed MU remodelling (Luff, 1998; Piasecki et al., 2016b) (Fig. 2). Furthermore, recent evidence suggests that a failure to reinnervate denervated fibres distinguishes sarcopenic from non-sarcopenic older men (Piasecki et al., 2018b), supporting the notion that this remodelling process occurs into older age but excessive fibre loss occurs when reinnervation can no longer sufficiently compensate for denervation. Thus, older muscle tends to comprise MU’s that are *fewer* in number and *larger* in size (in terms of fibre ratio) up to a certain point/age, when *fewer* and *smaller* MUs become more prevalent. Interestingly this remodelling process appears to be muscle specific, and more ‘successful’ in the TA compared to VL (Piasecki et al., 2018b). Given age-related alterations in MU size (increased fibre ratio) it is unsurprising to find that force steadiness (the ability to match a desired force) is impaired in older people (Dideriksen et al., 2012; Laidlaw et al., 2000), indicating there are important functional consequences of fibre loss and MU remodelling that reach beyond the loss of muscle size and strength.

Although the evidence strongly suggests an association between age associated MU remodelling and fibre loss, the proposed mechanisms are not entirely in agreement. Does the problem initially occur at the cell body in the spinal cord, somewhere along the axon, or does it originate within the myofibre, causing denervation and propagating along the alpha motor neuron in a retrograde manner? It is thus unclear if denervation is a cause or a consequence of fibre loss with further research clearly needed to address this.

### 3.3 Putative mechanisms of neuromuscular remodelling in older humans

The majority of the more detailed mechanistic data has been generated from rodent models (Deschenes, 2011; Gonzalez-Freire et al., 2014; Tintignac et al., 2015) and has focused on the neuromuscular junction (NMJ); the synapse between motor neuron and muscle fibre. The relationship of the NMJ and muscle fibre in this regard may be described in 3 stages. Firstly, with complete innervation, myonuclei close to the synapse express genes involved in NMJ maintenance (*MuSK*), which are suppressed in non-synaptic myonuclei. Secondly, with initial denervation proteasomal pathways are up regulated in all myonuclei. Thirdly, after prolonged denervation, there is an inhibition of autophagy and an increase in protein synthesis (via mTORC1) (Tintignac et al., 2015) (Fig. 2). Therefore a denervated fibre will immediately begin to atrophy, but will survive for an ill-defined amount of time, and these have been observed in a number of human biopsies (Lexell and Taylor, 1991; Spendiff et al., 2016; Zampieri et al., 2015). Further associations of age-related denervation and impaired reinnervation have been established in animal models, including alterations in oxidative stress (Jackson and Mcardle, 2016; Vasilaki et al., 2017), dysregulation of sterol metabolism in the nervous system (Pannérec et al., 2016), conversion of voltage-gated sodium channels on fibre membranes (Rowan et al., 2012) and a reduction in the number of key maintenance proteins such as PGC1-a (Gouspillou et al., 2013). As previously mentioned the number of SCs is decreased with age (Verdijk et al., 2014), and their involvement extends beyond the maintenance of the fibre; acting as a source of post-synaptic myonuclei their reduction results in reduced maintenance of this region and ultimately degeneration of the NMJ (Liu et al., 2017), and poor fibre regeneration following reinnervation (Dedkov et al., 2001). Additionally, terminal Schwann cells are implicated in the remodelling of MUs by initiating and guiding axonal sprouts, and are known to develop impairments with increasing age (Saheb-Al-Zamani et al., 2013) (Fig. 2). However, again - it is not entirely clear in all cases if these associations result of a cause or a consequence of denervation.

Furthermore, in many animal studies of this nature the measured response to denervation has followed nerve sectioning or ligation, therefore caution must be employed given that complete muscle denervation may promote the onset of different pathological pathways to that following repeated cycles of de/reinnervation of individual fibres.

In human studies, lifelong exercise has been suggested to minimise muscle loss (Mckendry et al., 2018) and prevent the age-related loss of MU number, and presumably fibre number in the TA of old (64 years) (Power et al., 2010) but not very old (79 years) athletes (Power et al., 2016). However a further study found masters athletes (69 years) had a similar number of MUs in the TA as age matched controls (Piasecki et al., 2016a). Although it is unlikely that exercise preserves the number of MU, it may improve the ability to reinnervate denervated fibres in order to preserve muscle fibre number, however this largely comes from biopsy studies which show increased fibre type grouping in master athletes (Zampieri et al., 2015). Although interesting, this grouping is indicative of a shift in fibre type composition (type I/II; an unbalanced ratio of fibre type composition will increase the probability of observing increased fibre type groupings) and does not directly prove the grouped fibres belong to the same MU. What is clear is that the notion of motor unit plasticity relating to the prevention of muscle fibre loss in humans is disproportionately underexplored and possible therapeutic targets in ageing (and diseases) warrants further investigation.

## 4 Conclusions

The present review details the major two influences upon loss of muscle mass and function with age: muscle fibre atrophy and muscle fibre loss. It is reasonably clear that both of these elements play a role in regulating muscle atrophy and dysfunction at the level of whole-muscle/groups. Nevertheless, few if any research groups focus upon both of these facets simultaneously in humans, nor the prospect of them being inter-related processes (denervation leading to atrophy and/or vice-versa). While this presents experimental challenges, only will investigating these processes simultaneously shed light on the mechanisms of human sarcopenia and dysfunction.

## Competing interests

The authors declare no conflict of interest.

## Funding

This work was supported by the Medical Research Council [grant number MR/P021220/1] as part of the MRC-ARUK Centre for Musculoskeletal Ageing Research awarded to the Universities of Nottingham and Birmingham, and the National Institute for Health Research, Nottingham Biomedical Research Centre.
